# Comparison of small extracellular vesicles isolated from plasma by ultracentrifugation or size-exclusion chromatography: yield, purity and functional potential

**DOI:** 10.1080/20013078.2018.1560809

**Published:** 2018-12-28

**Authors:** Kaloyan Takov, Derek M. Yellon, Sean M. Davidson

**Affiliations:** The Hatter Cardiovascular Institute, University College London, London, UK

**Keywords:** Exosomes, blood, plasma, vesicle purification, lipoproteins, endothelial cells, angiogenesis, endothelin-1

## Abstract

Interest in small extracellular vesicles (sEVs) as functional carriers of proteins and nucleic acids is growing continuously. There are large numbers of sEVs in the blood, but lack of standardised methods for sEV isolation greatly limits our ability to study them. In this report, we use rat plasma to systematically compare two commonly used techniques for isolation of sEVs: ultracentrifugation (UC-sEVs) and size-exclusion chromatography (SEC-sEVs). SEC-sEVs had higher particle number, protein content, particle/protein ratios and sEV marker signal than UC-sEVs. However, SEC-sEVs also contained greater amounts of APOB^+^ lipoproteins and large quantities of non-sEV protein. sEV marker signal correlated very well with both particle number and protein content in UC-sEVs but not in all of the SEC-sEV fractions. Functionally, both UC-sEVs and SEC-sEVs isolates contained a variety of proangiogenic factors (with endothelin-1 being the most abundant) and stimulated migration of endothelial cells. However, there was no evident correlation between the promigratory potential and the quantity of sEVs added, indicating that non-vesicular co-isolates may contribute to the promigratory effects. Overall, our findings suggest that UC provides plasma sEVs of lower yields, but markedly higher purity compared to SEC. Furthermore, we show that the functional activity of sEVs can depend on the isolation method used and does not solely reflect the sEV quantity. These findings are of importance when working with sEVs isolated from plasma- or serum-containing conditioned medium.

## Introduction

Extracellular vesicles (EVs) comprise a diverse population of nano- and microparticles released into the extracellular space by living cells and serving as paracrine, endocrine or autocrine signals [1,2]. Small EVs (sEVs), such as exosomes, have characteristic tetraspanin membrane markers (CD9, CD63, CD81) [3], are of predominantly endosomal origin [4–7] and have been shown to carry various proteins and nucleic acids [8–12].

EVs present in the blood have attracted the most interest as novel biomarkers for cancer, kidney, cardiovascular and neurodegenerative diseases [13] and they have also been shown to be functionally active, e.g. mediating cardioprotection [7,14–16], angiogenesis [17,18] and coagulation [19,20]. Blood plasma or serum sEVs can be isolated in large numbers [21,22] but ample evidence exists for the presence of a high degree of contaminants in vesicle isolates including non-EV proteins [22,23] and lipoproteins [21,24,25], regardless of the isolation method used [22,24,26,27]. Co-purification arises from similarities in size and/or density of the isolated sEVs and the co-existing lipoproteins or protein aggregates [28] or due to their physical association [23,24,28]. Despite the existence of multiple reports which compare sEV isolation methods [21–23,25,29–32], there is currently no agreement on an optimal technique to yield high-purity sEVs from blood.

In some cases, the non-EV protein contamination in vesicle samples obtained from plasma- or serum-containing fluids can be so extensive that specific sEV markers may even become undetectable in the isolates [25,31,33]. Additionally, in nearly 50% of the published experiments in which sEVs were isolated from cell culture-conditioned medium, the cells had been cultured in the presence of serum [34]. Considering that there are enormous numbers of lipoprotein particles in plasma and serum (~10^16^/ml) [28], this may result in a marked lipoprotein contamination in downstream experiments. It is therefore crucial to directly compare methods for sEV isolation in relation to contamination by both soluble protein and lipoproteins.

Ultracentrifugation (UC) is one of the most commonly used methods in the field [6,34], but reportedly leads to co-purification of lipoproteins [24] and soluble protein [23,25] with plasma sEVs. Instead, size-exclusion chromatography (SEC) has been proposed to provide sEVs with high purity [21,23,35,36] and higher functionality [31]. This is despite the fact that some lipoprotein classes (i.e. APOB^+^ lipoproteins) are known to co-isolate in large numbers with sEVs when using SEC [21,24,37,38]. Performing direct comparison of different techniques, e.g. UC versus SEC, is vital in order to establish an optimal method for plasma sEV isolation and to understand plasma sEVs characteristics.

The endothelium is the only organ in direct contact with blood and its diverse populations of sEVs. It is therefore of interest to determine the effect that plasma sEVs have on its function. It has been reported that EVs isolated from serum of healthy humans can activate endothelial cells and exhibit proangiogenic functions *in vitro* and *in vivo* [17,18]. Furthermore, erythrocyte, platelet and leukocyte EVs have all been shown to stimulate an angiogenic response (reviewed in Ref. [39]). However, there are no reports that combine a comparison of yield, purity and functional activity of plasma sEVs.

In this study, we compared sEVs isolated using the “gold standard”, UC (UC-sEVs) [6] or the increasingly popular technique of SEC (SEC-sEVs) [35], and analysed their yield, purity and promigratory effects on endothelial cells. We demonstrated that, despite a greater yield of SEC-sEVs, their purity is compromised when compared to UC-sEVs. We further showed that, while normalisation of sEVs to particle number or protein content is appropriate for UC-sEVs, this does not accurately represent the sEV content of all fractions from a SEC isolation. Plasma sEV isolates contained proangiogenic factors and promoted endothelial cell migration, but no correlation was found between migration levels and administered sEV content indicating that contaminating factors may also play a role in controlling the observed outcome. Thus, plasma sEVs obtained by UC appear to be of superior purity to those isolated by SEC while the choice of isolation method may strongly impact subsequent functional experiments.

## Materials and methods

### Ethical approval

All procedures contained within the application were approved by the Animal Welfare and Ethical Review Body (AWERB) and were conducted within the terms of the UK Home Office Guide on the Operation of Animals (Scientific Procedures) Act 1986, under Project Licence number PPL 70/8556 (“Protection of the Ischaemic and Reperfused Myocardium”). The investigation conforms to the guidelines from Directive 2010/63/EU of the European Parliament on the protection of animals used for scientific purposes or the NIH guidelines.

### Cell culture: human umbilical vein endothelial cells (HUVECs)

HUVECs were obtained from Lonza (Basel, Switzerland) as a pooled donor sample (C2519A). Cells were maintained in monolayers in Endothelial Cell Basal Medium 2 (C-22211, PromoCell, Heidelberg, Germany) supplemented with Endothelial Cell Growth Medium 2 SupplementPack (C-39211, PromoCell) in a conventional tissue culture incubator at 37°C/5% CO_2_. Cells were passaged using TrypLE Express Enzyme (12605028, ThermoFisher, Dartford, UK).

### Preparation of plasma or serum for UC or SEC

Male Sprague-Dawley rats (300–400 g) were obtained from Charles River (Margate, UK). Food and water were provided *ad libitum*. Blood sampling was performed at random times of the day. Rats were anaesthetised with 250 mg/kg pentobarbital. Thoracotomy was performed, and blood was collected from inferior vena cava in syringes pre-filled with citrate buffer (final concentration of ~15 mM after dilution with blood). No visible haemolysis was observed. Blood samples were centrifuged at 1600 g for 15 min, room temperature to remove cells. The supernatant plasma was transferred to new Eppendorf tubes and centrifuged at 10,000 g for 30 min, room temperature to remove debris and large vesicles. Plasma samples were used immediately or frozen at −80ºC.

### Isolation of plasma sEVs using UC

One millilitre (for characterisation experiments) or 4 ml (for functional experiments) plasma aliquot was diluted with PBS (Ca^2+^-free, Mg^2+^-free, 14190144, ThermoFisher) to ~7–8 ml and ultracentrifuged for 70 min at 100,000 g, 4ºC to pellet the sEVs (polycarbonate tubes, 355630, Beckman Coulter, Brea, USA; MLA-55 rotor, Optima MAX-XP, Beckman Coulter) according to Théry et al. [6]. Supernatant was discarded and sEVs were resuspended in PBS (~7–8 ml) for washing. A second UC run was performed for 70 min at 100,000 g, 4ºC. The sEV-rich pellet was resuspended to a final volume of 100–200 µl with PBS and frozen at −80ºC.

### Isolation of plasma sEVs using SEC

Commercially available qEVoriginal SEC columns (iZON Science, Oxford, UK) were used to fractionate blood plasma according to the manufacturer’s protocol [35]. One millilitre plasma aliquot was loaded on an SEC column and 0.5 ml fractions were collected as indicated (PBS used as eluent) and stored at −80°C. For maximising yield for functional experiments, sEV fractions 3.5–6.0 ml were pooled and concentrated using Vivaspin-4 filter (100 kDa, polyethersulfone membrane; Sartorius, Epsom, UK).

### Nanoparticle tracking analysis (NTA)

NTA was performed on a NanoSight LM10-HS instrument (NanoSight, Malvern, UK) using Violet (488 nm) laser module and NTA 3.1 software version. Particle concentration and size was determined following general recommendations [40] adapted to the type of samples in our studies. A syringe pump with constant flow injection was used and three videos of 30 s were captured with Camera Level of 15 and Detection Threshold of 4.

### Protein and nucleic acid content

BCA protein assay kit for low concentrations (ab207002, Abcam, Cambridge, UK) was used to quantify the protein content. The assay was performed according to the manufacturer’s instructions. The sample volumes used were 2–60 µl in final reaction volumes of 300 µl. The reactions were incubated for 120 min at 37°C. Absorbance was read at 562 nm on a FLUOstar plate reader (BMG Labtech, Aylesbury, UK). Protein concentrations were calculated using bovine serum albumin (BSA) standards and a four-parameter logistic curve.

Absorbance at 260 and 280 nm of early SEC fractions was also analysed using LVis plate and FLUOstar plate reader (BMG Labtech) to confirm the presence of nucleic acids and protein, respectively.

### Dissociation-enhanced lanthanide fluorescence immunoassay (DELFIA)

Specific markers of sEVs and lipoproteins were quantified using a previously described DELFIA [21,37] with modifications. Samples were added to a high-binding 96-well microplate (DY990, R&D Systems, Abingdon, UK). After overnight incubation at 4°C, blocking with 1% BSA/PBS for 1 h at room temperature was performed. This was followed by primary antibody incubation at 1 μg/ml in PBS for 2 h at room temperature (CD9: Clone M-L13, BD Biosciences, San Jose, USA; CD81: Clone JS-81, BD Biosciences; HSP70: Clone N27F3-4, Santa Cruz Biotechnology, Santa Cruz, USA; APOB: Clone H-300, Santa Cruz Biotechnology) and secondary antibody incubation at 0.25 µg/ml in PBS for 1 h at room temperature (biotin-conjugated goat anti-rabbit IgG for APOB, ab97073, Abcam or biotin-conjugated goat anti-mouse IgG1 for CD9, CD81 and HSP70, ab98691, Abcam). 1:1000 Eu-labelled streptavidin in DELFIA Assay Buffer (PerkinElmer, Beaconsfield, UK) was then added and incubated for 1 h at room temperature. Finally, 100 µl DELFIA Enhancement Solution (PerkinElmer) was added to each well and time-resolved fluorimetry was performed using a PHERAstar plate reader (BMG Labtech) with excitation at 337 nm, detection at 620 nm, integration start at 60 µs and integration time of 200 µs. Results are presented as arbitrary units (AU).

### Wes™ simple western

The presence of HSP70 and APOB in the isolates was confirmed using Wes™ Simple Western apparatus (ProteinSimple, San Jose, USA). ~0.4–0.6 µg protein was denatured using manufacturer-provided buffer (DTT based) and loaded in Wes™ multiwell plates. Protein separation, antibody binding and detection were performed using capillary cartridge separation and following manufacturer’s instructions. The following primary antibodies were used: HSP70 (at 10 μg/ml; clone N27F3-4, Santa Cruz Biotechnology), APOB (at 20 μg/ml; clone H-300, Santa Cruz Biotechnology) and alpha-Actinin-4 (at 32 μg/ml; clone C2C3, GeneTex, Irvine, USA). Anti-mouse (for HSP70; 042–205, ProteinSimple) and anti-rabbit (for APOB and alpha-Actinin-4; 042–205, ProteinSimple) secondary antibodies conjugated to horseradish peroxidase were used according to the manufacturer’s instructions. Images were obtained and analysed using Compass software for Simple Western (ProteinSimple).

### Protein arrays

Angiogenic factor profiling of UC-sEVs and SEC-sEVs was performed using Proteome Profiler Human Angiogenesis Array (ARY007; R&D Systems) following manufacturer’s instructions with some modifications. Each membrane was incubated with ~20 µg protein of UC-sEVs or SEC-sEVs (from functional experiments). Prior to incubation, sEVs were lysed with addition of 0.1% (v/v) Triton X-100 and vortexing for 30 s [41]. Streptavidin-DyLight 800 conjugate was used for detection of biotin-conjugated antibodies at 250 ng/ml (21,851, ThermoFisher). Membranes were imaged, and pixel densities were quantified on Odyssey system. The spot coordinates can be found on https://resources.rndsystems.com/pdfs/datasheets/ary007.pdf. Duplicate spot pixel densities were normalised to Reference Control spots and presented as relative pixel densities.

### Transmission electron microscopy (TEM)

TEM was performed by sEV labelling using 0.5% uranyl acetate following previously published protocols with modifications [6]. Briefly, ~2 µl of each sample (0.12–0.16 µg for samples containing high number of sEVs) was adsorbed on Formvar-carbon electron microscopy grids. After washing with H_2_O, the grids were transferred to a drop of uranyl acetate solution, pH 7 for ~3 min. Excess fluid was blotted, and the grids were imaged using a Jeol 1010 electron microscope (Jeol Ltd, Peabody, USA).

### EV-TRACK

The relevant data were submitted to the EV-TRACK knowledgebase (EV-TRACK ID: EV180051) [42].

### Endothelial cell migration

A modified Boyden’s chamber assay was performed to assess for promigratory functions of plasma sEV isolates on HUVECs following manufacturer’s instructions with some modifications (NeuroProbe, Gaithersburg, USA). Bottom wells of a 12-well NeuroProbe chemotaxis chamber (AA12, NeuroProbe) were filled with vehicle (PBS), 10% FBS (positive control) and SEC- or UC-isolated sEVs as indicated (*n* = 5). Eight micrometres polycarbonate track-etch membranes (PFB8, NeuroProbe) were used as filters for HUVEC migration. Thirty-thousand HUVECs/well in Endothelial Serum-Free Defined Medium (Cell Applications Inc, San Diego, USA; 113–500, Sigma) were plated in the top wells and the chamber was incubated for 6 h at 37°C, 5% CO_2_. At the end of the incubation period, membranes were collected, and the top side was scraped to remove non-migrated cells. Membranes were fixed in 100% cold methanol, stained using 0.5 % (w/v) Crystal Violet solution and scanned using CanoScan LiDE 220 scanner (Cannon). ImageJ was used to quantify the total staining intensity of each well. Intensities were measured for duplicate wells and normalised to the positive control (10% FBS). Representative images of each group were acquired on Nikon Eclipse TE200 inverted microscope (Nikon).

### Statistical analysis

Data are plotted as mean ± SEM. GraphPad Prism was used for statistical analyses and graph production (GraphPad Software, San Diego, USA). Pearson’s or Spearman’s correlation tests were performed where indicated after a Kolmogorov-Smirnov test for normality. Statistical comparisons were performed using Student’s *t*-tests or one-way ANOVA with Tukey’s post-hoc test as indicated. *p* value of < 0.05 was considered significant.

## Results

### Particle and protein content of plasma sEV samples isolated by UC or SEC

Platelet-free plasma was isolated from the blood of rats in accordance with published recommendations [26,43]. In order to directly compare protein and particle yields, equal volumes of blood plasma collected from the same animal were processed using differential UC [6] or commercially available SEC columns (qEV, iZON) (Supplementary Figure 1(a)).

**Figure 1.**
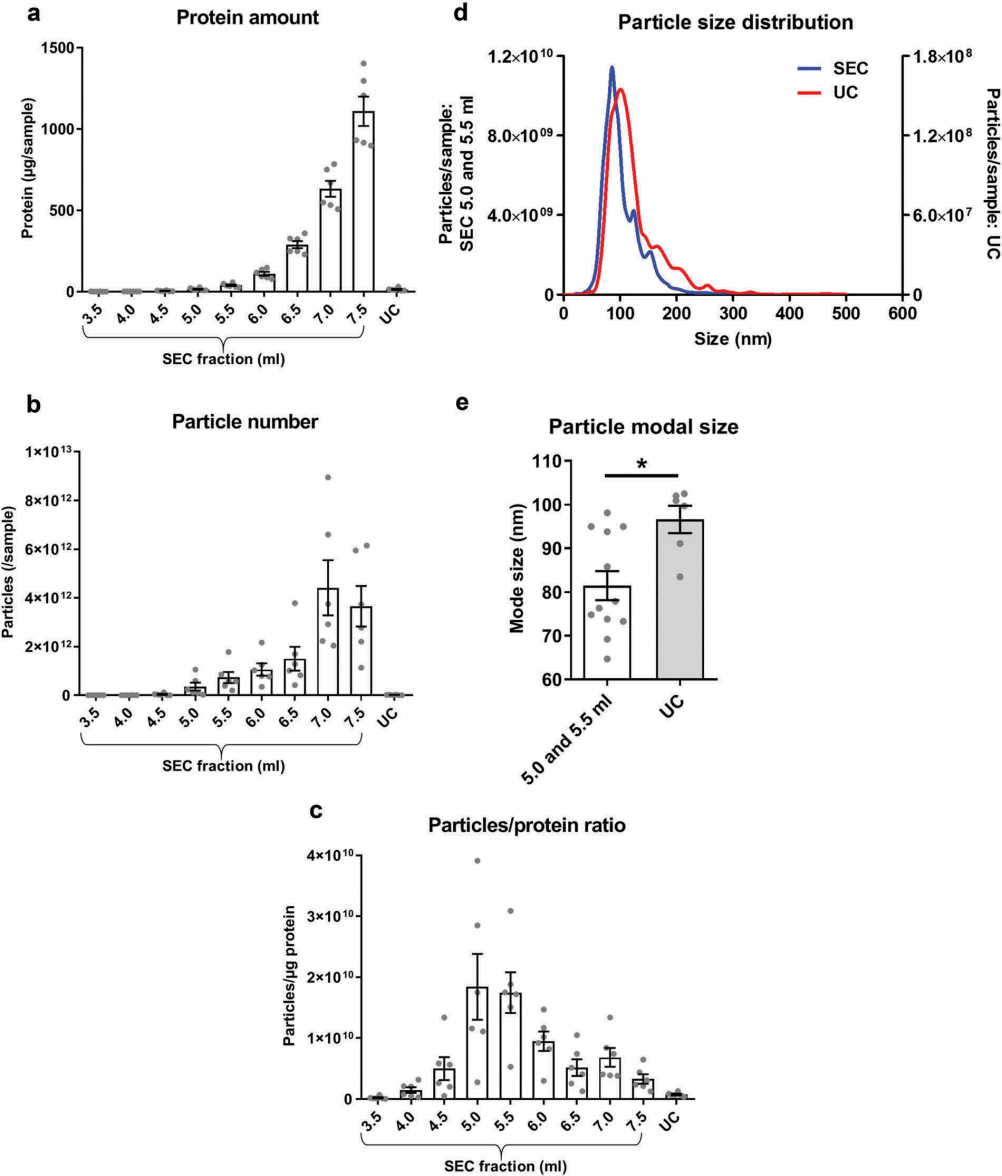
Protein and particle content in samples obtained from rat blood plasma using size exclusion chromatography (SEC) or ultracentrifugation (UC). Protein content was measured by BCA assay and particle concentration and size by NTA. (a): Protein amount in SEC fractions 3.5–7.5 ml and UC samples. *n* = 6. (b): Particle number in SEC fractions 3.5–7.5 ml and UC samples. *n* = 6. (c): Particle/protein ratio for SEC fractions 3.5–7.5 ml and UC samples. *n* = 6. (d): Particle size distribution for SEC fractions 5.0–5.5 ml and UC demonstrates the presence of particles in the typical sEV range. SEC fractions 5.0 and 5.5 ml were used due to their high particle/protein ratio. *n* = 6–12, curve shows mean values. (e): Modal size of the particles isolated from SEC (5.0–5.5 ml) and UC. *p < 0.05; Student’s *t*-test. *n* = 6–12.

Pilot SEC experiments (Supplementary Figure 1(b)) and our previous studies [37] indicated that sEVs from plasma samples eluted with a peak in SEC fractions ~5–6 ml while the bulk of protein appeared at ~10 ml. Hence, subsequently, fractions of 3.5–7.5 ml from SEC were collected and analysed. As expected, protein content gradually increased with each fraction (Figure 1(a)). Strikingly, the number of particles also followed a similar pattern without an obvious early peak (5–6 ml) as measured by NTA (Figure 1(b)). The ratio of the number of particles to the protein content (particle/protein ratio) has previously been suggested to be an adequate marker of sEV purity [22]. This value peaked at SEC fractions 5.0–5.5 ml (Figure 1(c)) indicative of an enrichment of particles relative to protein in these fractions. Therefore, these fractions were subsequently referred to as “SEC-peak”.

The particle content of the UC samples was negligible compared to SEC-peak, and protein content and particle/protein ratios were also lower in UC samples than SEC-peak fractions (Figure 1(a–c)). Although the size distribution of particles in the SEC-peak and UC samples appeared similar (Figure 1(d)), particle modal size in the UC samples was significantly higher than SEC-peak (UC: 96.6 ± 3.1 nm vs SEC-peak: 81.5 ± 3.3 nm, *p* < 0.05; Figure 1(e)).

Overall, based on protein content and particle size distribution analysis, a higher particle yield and greater apparent purity, based on particle/protein ratios, is achieved with SEC compared to UC.

### Characterisation of sEV content and purity of UC and SEC samples

Particle number and protein content are not sufficient to completely understand sEV yield and purity, since NTA does not distinguish between sEVs and similarly sized molecules such as small lipoproteins. To address this issue, we used DELFIA (a high-sensitivity immunoassay [21,37]), to analyse content of sEVs markers (CD81 and HSP70) [3,14,44] and contaminant lipoproteins (APOB) [24,28,31].

**Figure 2.**
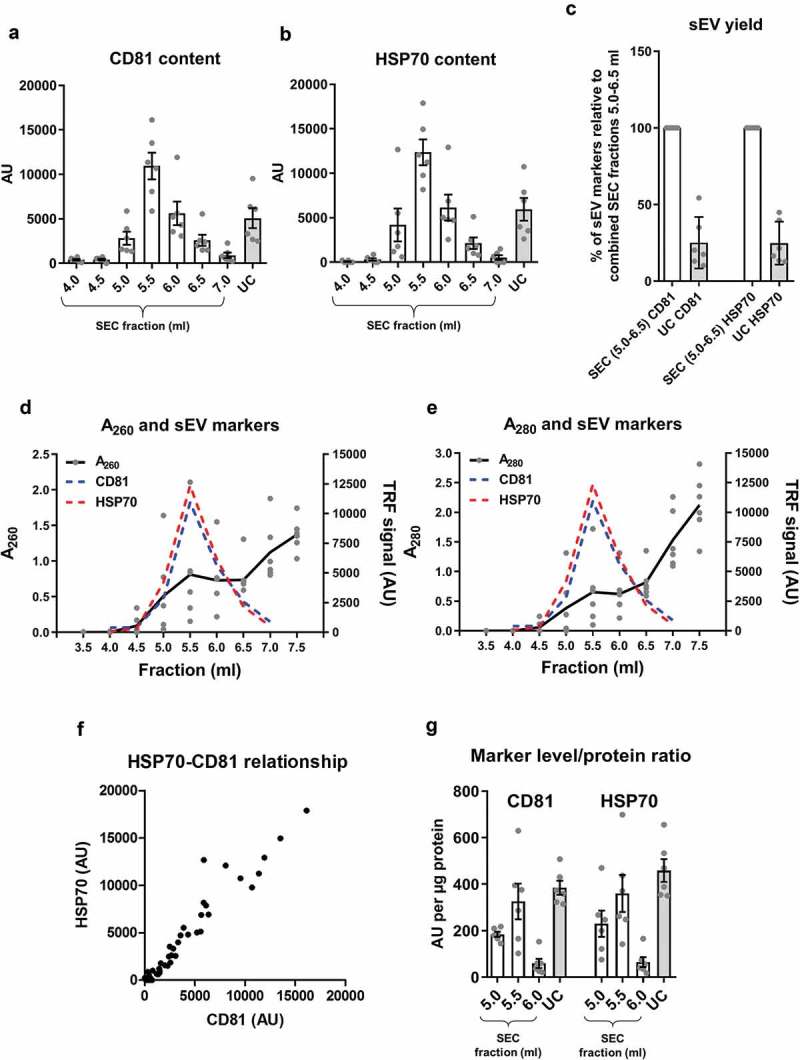
sEV content, as determined by the expression of marker proteins, in rat blood plasma samples obtained using SEC or UC. (a,b): sEV (CD81 – panel (a), HSP70 – panel (b)) markers were measured by DELFIA for SEC fractions 4.0–7.0 ml and UC samples. AU – arbitrary units normalised to volumes in each sample. *n* = 6. (c): UC sEV yield presented as a % of combined SEC fractions 5.0–6.5 ml CD81 and HSP70 signal (data from (a) and (b)). *n* = 6. (d,e): Absorbance of SEC samples at 260 nm (A_260_), representing relative nucleic acid concentrations; and at 280 nm (A_280_), representing relative protein concentrations. Note the presence of early peaks at both A_260_ and A_280_ which coincide with the CD81 and HSP70 marker signal peaks (data from panels (a) and (b), dashed lines). *n* = 6. (f): Correlation between HSP70 and CD81 signal. SEC fractions 4.0–7.0 ml and UC samples included. *p* < 0.0001; Spearman’s correlation test, Spearman *r* = 0.958. *n* = 48. (g): sEV marker signal (from panels (a) and (b)) was normalised to total protein amount for the peak sEV SEC fractions 5.0–6.0 ml as well as for the UC samples. *n* = 6.

Large quantities of CD81 and HSP70 were detected in SEC fractions 5.0–6.5 ml (with a clear peak at 5.5 ml; Figure 2(a–c)), suggestive of high sEV content. Nucleic acid and protein content can be determined by measuring the samples’ absorbance at 260 and 280 nm, respectively. This confirmed the presence of an early peak at 5.5 ml for both A_260_ and A_280_ coinciding with the CD81 and HSP70 peaks (Figure 2(d,e)). UC samples contained CD81^+^ and HSP70^+^ particles, but the yield was only ~25% of the combined sEV-containing SEC fraction yield (Figure 2(a–c)). The performance of SEC columns was found to be highly consistent between experiments (Supplementary Figure 2) and a strong positive correlation between CD81 and HSP70 signal in individual sEV isolates was found (*p* < 0.001), confirming the efficient isolation of vesicles positive for both CD81 and HSP70 (Figure 2(f)). Interestingly, despite the lower content of particles and CD81 and HSP70 markers in UC isolates (Figure 1(b,c), 2(c)), the sEV marker/total protein ratio was generally higher for UC samples compared to peak sEV SEC fractions (Figure 2(g); also confirmed using pooled SEC fractions: see Figure 8(e)).

**Figure 3.**
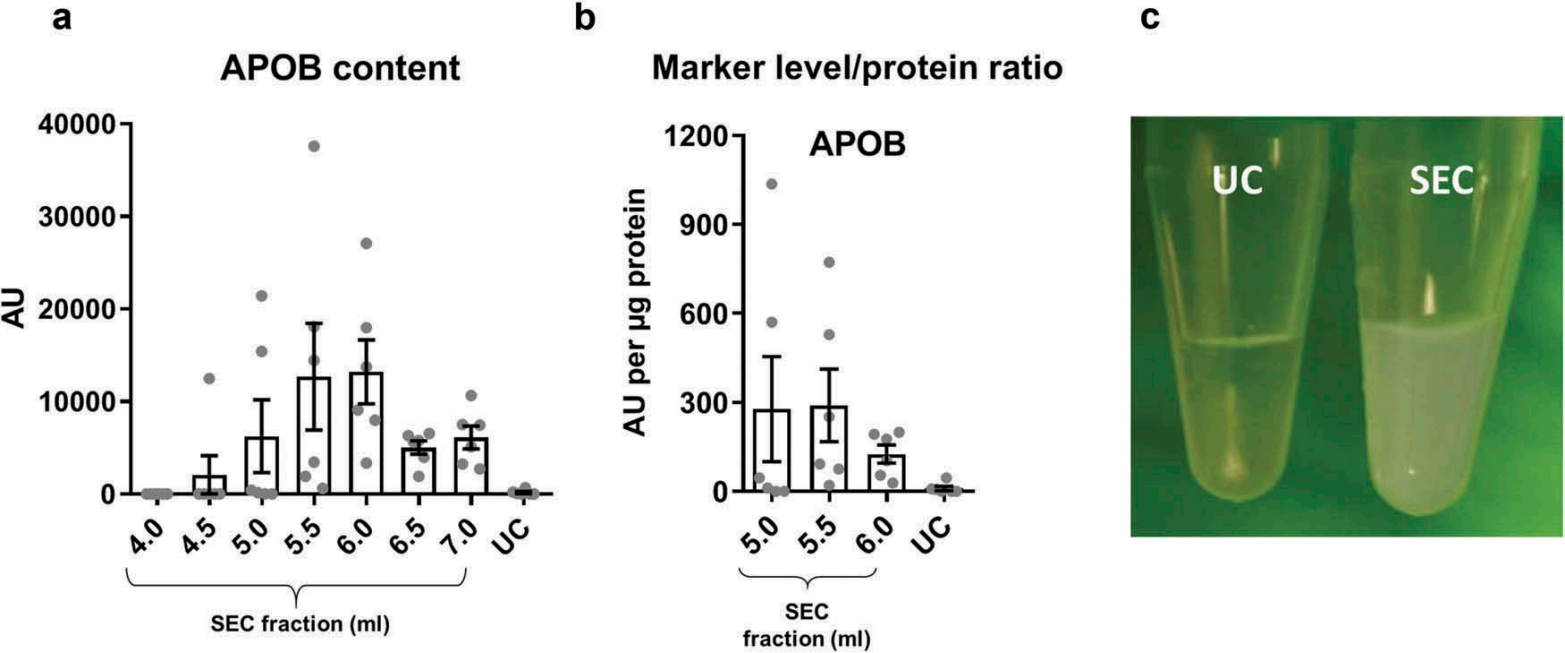
Lipoprotein content in samples of rat blood plasma obtained with SEC or UC. (a): APOB as a marker of lipoproteins measured by DELFIA for SEC fractions 4.0–7.0 ml and UC samples. AU – arbitrary units normalised to volumes in each sample. *n* = 6. (b): APOB lipoprotein marker signal was normalised to total protein amount for the peak sEV SEC fractions 5.0–6.0 ml as well as for the UC samples. *n* = 6. (c): UC sample visual appearance compared to pooled and concentrated SEC fractions 3.5–6.0 ml. Note the opaque appearance of the SEC sample indicative of the presence of lipids.

**Figure 4.**
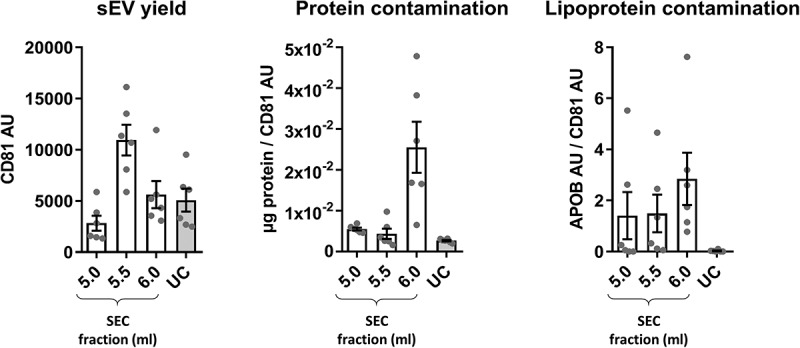
Summary of yield and purity of sEVs isolated by SEC or UC. Summarised key points from Figures 1 to 3. sEV yield (left panel, see Figure 2(a)), protein contamination (middle panel, see Figure 2(g)) and lipoprotein contamination (right panel, see Figures 2(a) and 3(a) shown for the peak sEV fractions (5.0, 5.5 and 6.0 ml) of SEC and UC samples. CD81 signal was used as an estimate of sEV content.

**Figure 5.**
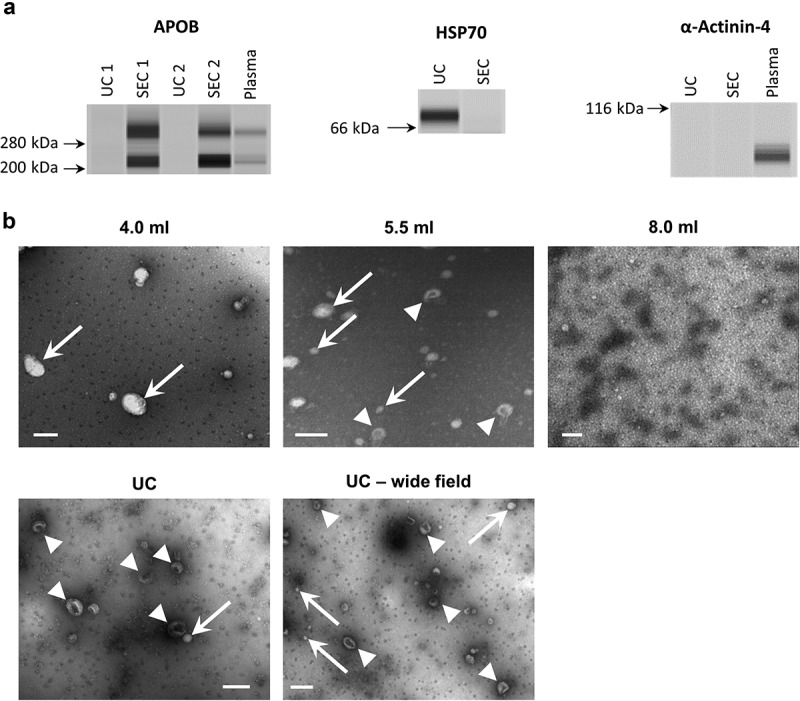
Western blotting and electron microscopy of rat blood plasma samples obtained with SEC or UC. (a): Wes™ Simple Western (ProteinSimple; see Methods for details) for APOB (left), HSP70 (right, top), alpha-Actinin-4 (right bottom) of UC and pooled SEC (3.5–6.0 ml) samples. (b): TEM images of SEC fractions 4.0, 5.5 and 8.0 ml (top panels) and UC-sEVs (bottom panels). Arrows indicate lipoprotein-resembling structures. Arrowheads indicate sEVs. Scale bar: 200 nm.

**Figure 6.**
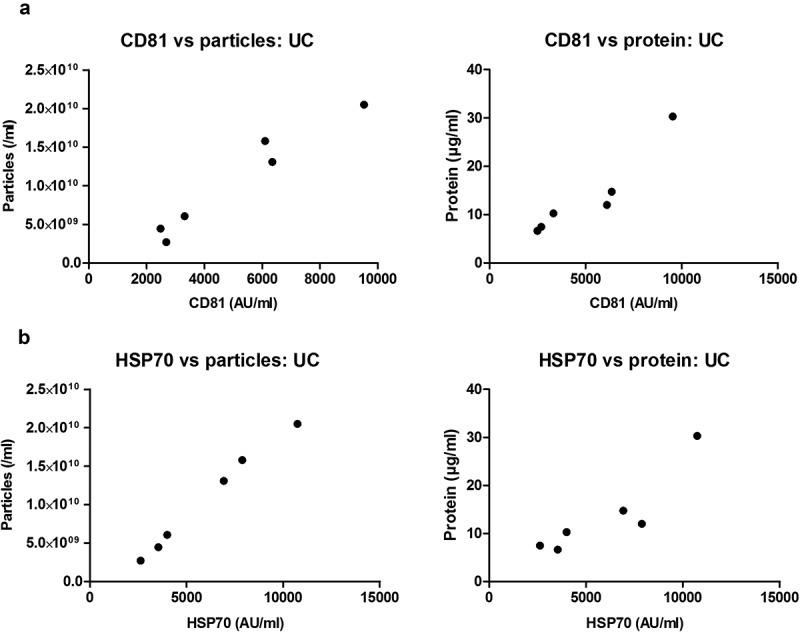
Correlation of sEV marker signal and particle or protein concentration of vesicles isolated by UC of rat blood plasma. Particle (left panels) or protein (right panels) concentration of UC samples plotted against CD81 (a) or HSP70 (b) marker signal. Positive correlations were found for all four panels ((a): CD81 vs particles: *p* < 0.001; Pearson’s correlation test, Pearson *r* = 0.975. CD81 vs protein: *p* < 0.01; Pearson’s correlation test, Pearson *r* = 0.938. (b): HSP70 vs particles: *p* < 0.0001; Pearson’s correlation test, Pearson *r* = 0.996. HSP70 vs. protein: *p* < 0.05; Pearson’s correlation test, Pearson *r* = 0.899).

**Figure 7.**
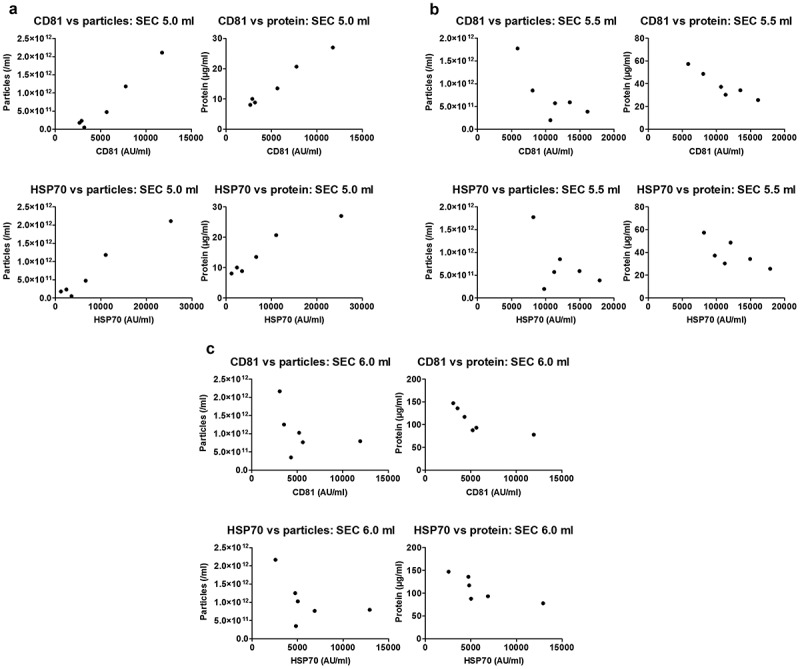
Correlation of sEV marker signal and particle or protein concentration of vesicles isolated by SEC of rat blood plasma. Particle (left panels) or protein (right panels) concentration of peak sEV-SEC fractions 5.0 ml (a), 5.5 ml (b) and 6.0 ml (c) plotted against CD81 (top panels) or HSP70 (bottom panels) marker signal. Positive correlations were found for all four panels in (a) (CD81 vs particles: *p* < 0.001; Pearson’s correlation test, Pearson *r* = 0.983. CD81 vs protein: *p* < 0.001; Pearson’s correlation test, Pearson *r* = 0.990. HSP70 vs. particles: *p* < 0.001; Pearson’s correlation test, Pearson *r* = 0. 0.978. HSP70 vs. protein: *p* < 0.01; Pearson’s correlation test, Pearson *r* = 0.963). No positive correlations were found in panels (b) and (c) (*p* > 0.05; Pearson’s or Spearman’s correlation test as required, except top right panels where CD81 signal and protein concentration correlated negatively, *p* < 0.01; Pearson’s correlation test, Pearson *r* = −0.937 for top right panel in (b) and *p* < 0.05; Spearman’s correlation test, Spearman *r* = −0.943 for top right panel in (c)).

**Figure 8.**
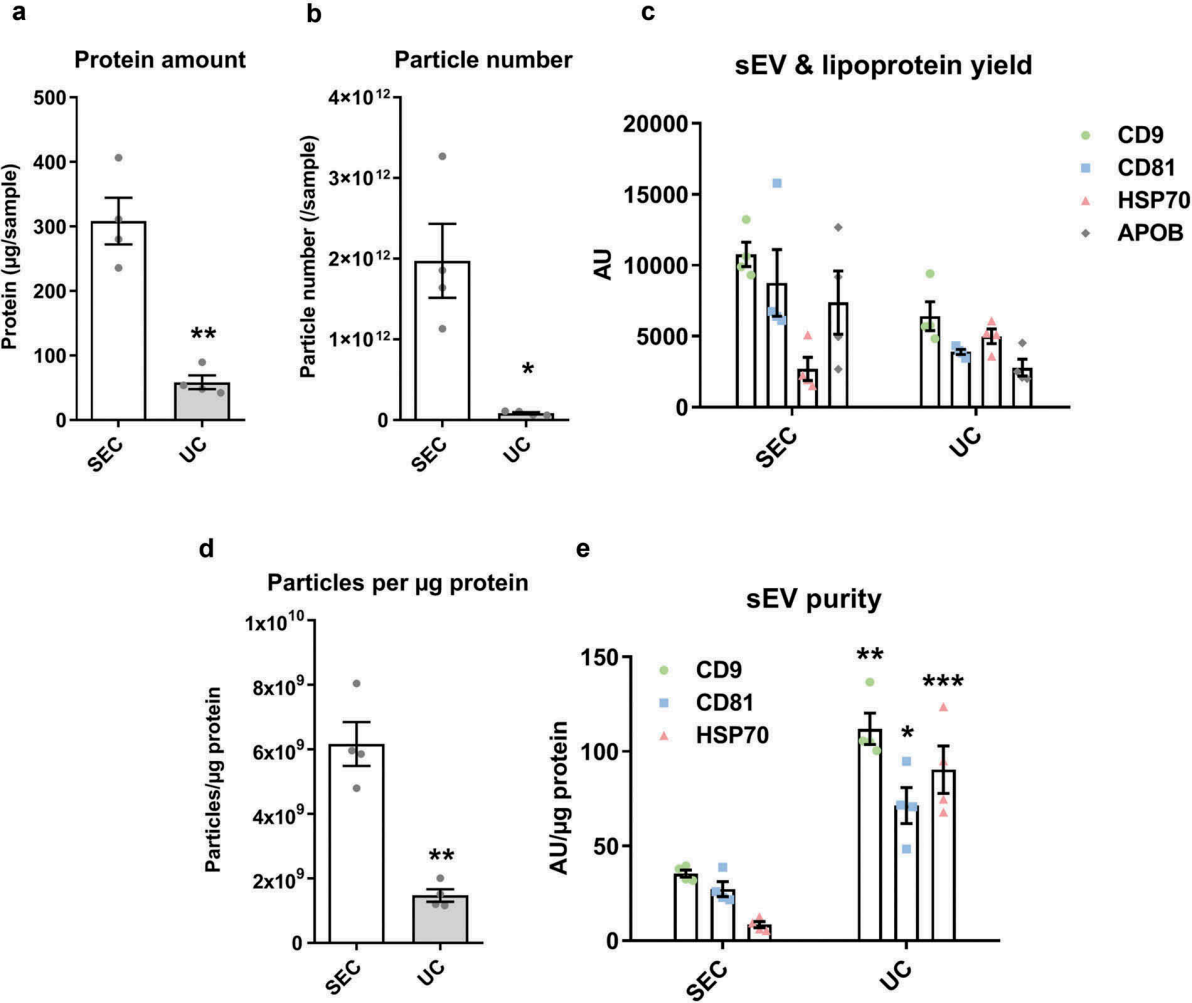
Characterisation of sEVs isolated by SEC or UC for use in endothelial cell migration experiments. For SEC isolation of sEVs, 1 ml of plasma was loaded on a SEC column and fractions 4.0–6.0 ml were collected, pooled and concentrated on Vivaspin-4 ultrafiltration units (100 kDa cut-off). For UC isolation of sEVs, 4 ml of starting plasma volume were used. (a): Protein amount in SEC and UC samples measured by BCA assay. *n* = 4. **p < 0.01. (b): Particle number in SEC and UC samples measured by NTA. *n* = 4. *p < 0.05. (c): sEV (CD9, CD81, HSP70) and lipoprotein (APOB) markers measured by DELFIA for SEC and UC samples. AU – arbitrary units normalised to volumes in each sample. *n* = 4. Note the marker levels were also normalised to starting plasma volumes and represent the yield of sEV and lipoproteins from 1 ml plasma. (d): Particle/protein ratio for SEC and UC samples. *n* = 4. **p < 0.01. (e): sEV marker signal normalised to total protein amount for SEC and UC samples as a measure of purity of sEVs from soluble protein. Note the higher sEV/total protein content in UC samples despite the lower particle to protein ratio (panel (d)). **p < 0.01 UC CD9 vs. SEC CD9, *p < 0.05 UC CD81 vs. SEC CD81 and ***p < 0.001 UC HSP70 vs. SEC HSP70, Student’s *t*-test.

Next, we compared the degree of contamination with APOB^+^ lipoproteins when using the two techniques. APOB^+^ lipoproteins were detected in the sEV isolates of both techniques but levels in the peak sEV SEC fractions (5.0–6.0 ml) were markedly higher indicative of substantial lipoprotein contamination in SEC isolates (Figure 3(a)). Moreover, normalisation of APOB signal to protein content showed a striking ~30 times greater quantity of APOB in the peak sEV fraction of SEC (5.5 ml) compared to the UC samples (Figure 3(b)). Pooling and concentrating the peak sEV fractions of SEC resulted in a visually opaque emulsion suggestive of a marked presence of lipids (Figure 3(c)). Additionally, normalisation of APOB signal to CD81 content, as an estimate of sEV purity from lipoproteins, also demonstrated almost 60 times higher APOB/CD81 ratio in the peak sEV fraction of SEC (5.5 ml) compared to the UC samples (Figure 4).

Collectively, these data indicate that SEC resulted in a higher yield of sEVs but with marked contamination by soluble protein and lipoproteins. The key data on yield and purity are summarised on Figure 4.

Western blotting confirmed the results obtained with DELFIA (Figure 5(a)). The sEV marker HSP70 was more abundant in UC-sEVs while the lipoprotein marker APOB was markedly higher in pooled SEC-sEVs (Figure 5(a)). As expected, alpha-Actinin-4 (a marker of large- and medium-sized EVs [3]) was virtually absent in the sEV isolates while it was present in non-purified plasma (Figure 5(a)).

TEM can be used to confirm the contents of the isolates. When dried for staining, lipoproteins have a typical, round white appearance, while EVs take on a collapsed vesicle or so-called “cup-shaped” appearance [45]. Representative TEM images supported the nanoparticle tracking and immunoassay data obtained for both UC and SEC samples (Figure 5(b)). Pre-sEV fractions of SEC contained predominantly larger, round, lipoproteins-like structures, while the peak sEV fraction 5.5 ml contained sEVs in addition to many particles resembling lipoproteins (Figure 5(b), upper panels). The late, post-sEV fractions comprised mainly of dense protein and lipoprotein material (Figure 5(b), upper panels). TEM images of UC-sEVs showed mostly sEVs with occasional presence of lipoprotein-like particles (Figure 5(b), lower panels).

### Particle and protein content as estimates of sEV yield

To conduct functional or analytical experiments, sEV isolates are usually normalised either in terms of equal particle number or protein concentration. To assess the ability of these approaches to correctly estimate the sEV content in plasma isolates, sEV marker content was plotted against particle number or total protein concentration. For UC samples, a strong positive correlation was found between particle number and CD81 or HSP70 content (*p* < 0.001 and *p* < 0.0001, respectively; Figure 6(a,b)), and between protein concentration and CD81 or HSP70 content (*p* < 0.01 and *p* < 0.05, respectively; Figure 6(a,b)). Strikingly, however, particle number or protein content did not positively correlate with sEV markers for all SEC fractions (Figure 7(a–c)). Positive associations were found only in fraction 5.0 ml for particle number and CD81 or HSP70 content (*p* < 0.001, Figure 7(a)) and protein concentration and CD81 or HSP70 content (*p* < 0.001 and *p* < 0.01, respectively; Figure 7(a)). Meanwhile, neither particle number nor protein concentration of peak sEV SEC fractions 5.5 and 6.0 ml showed a positive correlation with sEV markers (Figure 7(b,c)).

Overall, estimation of the amount of isolated plasma sEVs by total protein content or particle number is appropriate for UC-isolated sEVs and some fractions of the SEC-isolated sEVs but may not be a suitable approach for all peak sEV-SEC fractions.

### Comparison of the UC-sEVs and SEC-sEVs function using an endothelial cell migration assay

For functional analysis, sEVs from different samples can be normalised either by particle number, protein content or sEV marker content. We investigated whether these different methods can influence results in an endothelial cell migration assay.

sEV yield was maximised for functional experiments using larger starting plasma volume for UC or concentration of sEV-containing fractions for SEC. Particle, protein, lipoprotein and sEV marker data were in accordance with Figures 1–3 (Figure 8(a–e)).

**Figure 9.**
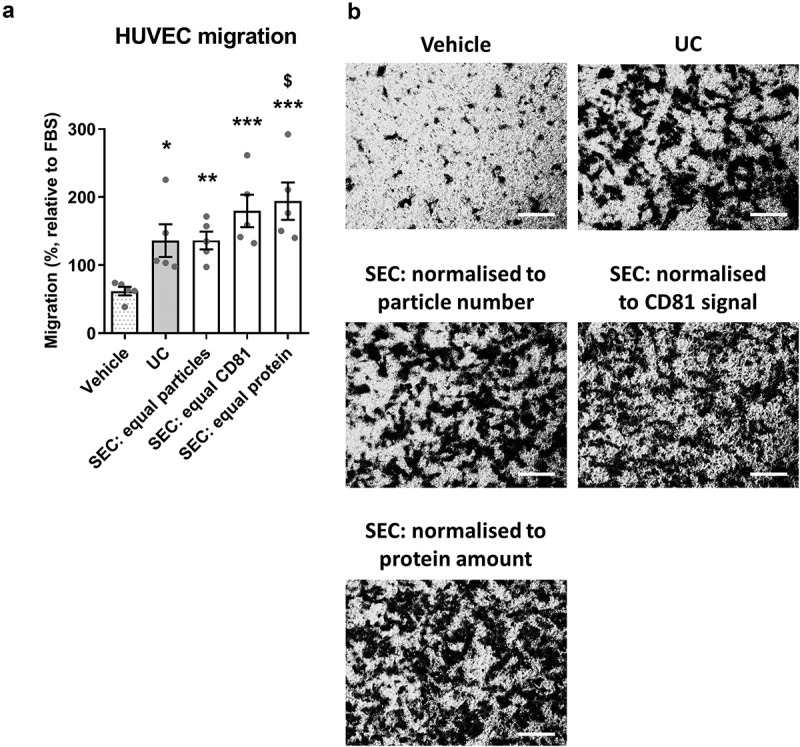
SEC-sEVs and UC-sEVs promote migration of endothelial cells *in vitro.* (a) modified Boyden’s Chamber assay was employed to study HUVEC migration in response to SEC- and UC-isolated sEVs. 1 × 10^10^ particles/ml were used for the UC group. SEC-isolated sEVs were adjusted to match the UC sample in terms of particle number (“SEC: equal particles”), CD81 content (“SEC: equal CD81”) or protein content (“SEC: equal protein”). Vehicle group contained PBS. Relative levels of SEC-sEV dose-response are: “SEC: equal protein” = ~4× “SEC: equal particles” and “SEC: equal CD81” = ~11× “SEC: equal particles” (a): All groups showed higher HUVEC migration than Vehicle control (*p < 0.05, UC vs Vehicle; **p < 0.01, SEC: equal particles vs. Vehicle; ***p < 0.001, SEC: equal CD81 vs. Vehicle and SEC: equal protein vs. Vehicle, one-way repeated measures ANOVA with Tukey’s post-hoc test, *n* = 5). SEC: equal protein induced more HUVEC migration than the UC group ($ *p* < 0.05, one-way repeated measures ANOVA with Tukey’s post-hoc test, *n* = 5). Data are presented as whole-well staining intensities normalised to a positive control (10% FBS). (b): Representative microscopy pictures confirming the data shown in (a). Scale bar: 200 µm.

For functional studies, Boyden’s Chamber migration experiments were conducted with endothelial cells (HUVECs). Administration of UC-sEV stimulated HUVEC migration (Figure 9(a,b)). An equivalent particle number of SEC-sEVs promoted migration of endothelial cells to a similar degree (Figure 9(a,b)) despite the CD81 signal being ~11 times lower in SEC samples (Figure 8(d,e)). Interestingly, loading much higher particle numbers of SEC-sEVs, so as to match the CD81 signal of the UC-sEVs, led to only a fairly small, non-significant increase in HUVEC migration (Figure 9(a,b)). Finally, equalising SEC-sEVs based on protein concentration produced a significantly higher migration with SEC-sEVs despite there being approximately three times less CD81 (Figures 8(d,e), 9(a,b)).

**Figure 10.**
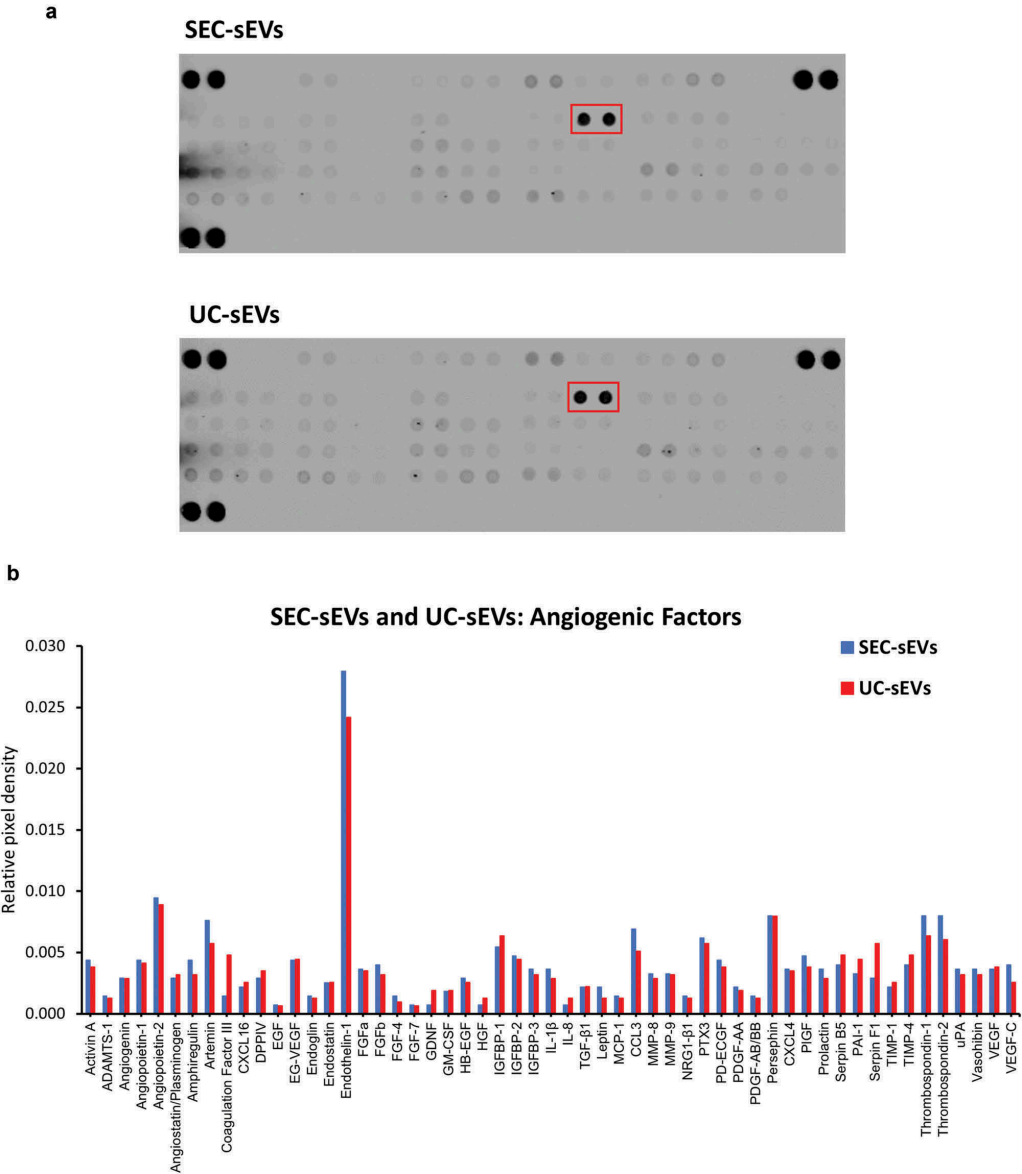
Angiogenic factors present in SEC-sEVs and UC-sEVs isolates. ~20 µg protein of SEC-sEVs or UC-sEVs samples isolated for functional studies (see Figure 6 and Supplementary Figure 3) were incubated on ARY007 protein array membranes (R&D Systems). Relative levels of 55 angiogenesis-related factors were quantified and presented. (a): Array membrane images. The red box indicates the most abundant protein – endothelin-1. (b): Quantification of panel (d). Relative pixel densities represent mean duplicate spot pixel densities normalised to Reference Control spots.

To obtain a preliminary profile of angiogenic factors present in the plasma sEV samples which may be responsible for the promigratory effects, protein arrays were used. Approximately twenty micrograms protein of SEC-sEVs or UC-sEVs were incubated on protein array membranes (Figure 10(a)). Multiple potential promigratory factors were found to be present in the sEV isolates with the most abundant one being endothelin-1, a known stimulator of endothelial cell migration [46,47] (Figure 10(a,b)). Further studies will be required for precise identification of the factor(s) and downstream mechanisms responsible for promigratory effects of plasma sEVs on endothelial cells.

Overall, our data showed that plasma sEVs promote migration of endothelial cells but normalisation of sEV samples to particle number, protein content or sEV marker content can lead to dramatically different functional outcomes. Protein profiling identified multiple candidates which may be responsible for the observed effects with endothelin-1 being the most abundant proangiogenic factor present in sEV isolates. We also observed a lack of direct association between the extent of endothelial cell migration and added sEVs indicating that the effect of the isolates may be influenced by contaminating, non-vesicular material.

## Discussion

In this report, UC and SEC were compared as methods for purifying sEVs from blood plasma. Higher yields of sEVs were obtained with SEC, but samples had substantially compromised purity in comparison to UC. Particle and protein content alone were insufficient to provide an accurate estimation of sEV content or lipoprotein contamination in a plasma vesicular isolate. This confirmed the importance of measuring the levels of specific sEV markers and contaminating factors. APOB content analysis suggested that contaminating lipoproteins were present in excess in the SEC-sEV samples. Furthermore, sEV marker content relative to total protein was lower in peak sEV-SEC fractions compared to the UC-sEVs indicating that soluble protein may also be a contaminating factor in SEC isolates. Finally, using an endothelial cell migration assay, we demonstrated that sEV functional outcomes are dependent on the isolation method used and effects of blood plasma sEVs may be obscured by the presence of contaminating factors.

It has been previously argued that a pure population of 10^10^ sEVs theoretically contains ~1 µg of protein [7]. Other studies have suggested that even higher ratios of particles per µg of protein have to be present in order to consider a population of sEVs highly pure [22]. Nevertheless, with plasma or serum isolates, it is virtually impossible to achieve such purities [22]. Intriguingly, we found that particle/protein ratio is not an accurate measure of purity in blood-derived sEV samples. Analysing SEC isolation as a standalone method showed very high particle/protein ratios in SEC-sEV fractions, indicative of high purity. Moreover, when SEC was systematically compared to UC, particle/protein ratios obtained for peak sEV fractions from SEC were markedly higher than UC values suggesting SEC-sEVs are of better purity than UC-sEVs. In contrast, immunoassays for APOB^+^ lipoproteins and sEV markers indicated the higher particle number in SEC-sEVs did not represent bona fide sEVs. Indeed, as previously reported by others, a huge number of plasma particles detected by NTA are not sEVs [21]. Overall, taking our data and previous considerations into account, particle/protein ratio is not valid as a sole indicator for purity of plasma sEV isolates.

Importantly, we showed that total APOB, as a marker of various lipoproteins [48], is markedly higher in SEC compared to UC samples. These findings are in accordance with our previous studies [37] and other reports which have demonstrated that lipoproteins are likely co-isolated with sEVs when using plasma [21,24,38]. Using commercially available SEC columns, Sodar et al. showed that APOB^+^ events co-purify with sEV marker^+^ events [24], while Welton et al. used an in-house prepared SEC column and also demonstrated co-isolation of APOB^+^ material with CD9^+^/CD63^+^/CD81^+^ particles [21]. Therefore, the co-purification of APOB^+^ lipoproteins and sEVs using SEC is likely to be an inherent property of SEC as a method for plasma vesicle isolation.

Another indicator of lipoprotein contamination of SEC-sEVs is the modal size of the particles which was lower in SEC-sEVs than UC-sEVs. This may be due to the presence of more, smaller lipoprotein particles in SEC samples (e.g. APOB^+^ low-density lipoproteins [24]) which could reduce the modal size significantly. Notably, an alternative explanation is that sEVs might be aggregated or fused after UC due to the high centrifugation speed used [49]. This might be expected to influence downstream functional effects but we have previously used UC to isolate plasma sEVs and found that they remain functionally active in being able to protect both primary cardiomyocytes and intact hearts from ischaemia and reperfusion injury [14], and in this report, we demonstrate that they promote migration of endothelial cells. This size difference between SEC-sEVs and UC-sEVs could also be confirmed using additional methods, e.g. vesicle sizing using TEM images [50].

In another study using UC and SEC for isolation of sEVs from rat blood plasma, Baranyai et al. found that both methods lead to significant protein impurities in the sEV isolates [23]. It is important to note that authors used only albumin as a surrogate marker for protein contamination [23], while in this study we used more general total protein content alongside APOB as marker for lipoprotein contamination. Additionally, our study has the advantage of using equal plasma volumes from the same animal for each UC and SEC biological replicate which allowed us to directly compare contaminating factors in the same experiment. In accordance with albumin data in Baranyai et al. [23], our results indicated that peak sEV fractions isolated by SEC are likely contaminated with soluble protein. Interestingly, the direct comparison to UC demonstrated that this soluble protein is generally higher for SEC-sEVs than UC-sEVs as shown by normalisation of sEV signal to total protein content. This is a further advantage of UC over SEC for plasma sEV purification.

Similarly to other authors [21], we found that NTA or protein content may not be good parameters to estimate the number of sEVs for most peak sEV SEC fractions. This is clearly demonstrated by the lack of correlation between particle number or protein content and CD81 or HSP70 levels for peak sEV SEC fractions 5.5 ml and 6.0 ml. On the other hand, UC samples and SEC fraction 5.0 ml showed very good positive correlation for CD81 or HSP70 and particle number or protein content. These observations suggest that a higher particle number or protein level correspond to a higher sEV content for UC samples, but this is not necessary the case for all SEC fractions from an isolate. Furthermore, the only fraction from SEC which shows very good association of increasing particle number or protein content with higher sEV markers (i.e. 5.0 ml) demonstrates lower purity from proteins and marked contamination with lipoproteins. This questions the validity of using these indices as a means of general normalisation of SEC-sEVs treatments for functional experiments and indicates that normalisation parameters should be carefully selected, and their choice justified.

Other particle enumeration methods may also lead to similar inaccuracies in the estimation of the actual vesicle counts (e.g. tuneable resistive pulse sensing is majorly influenced by lipoproteins [24]). Nevertheless, some techniques, such as flow cytometry, may be better able to distinguish vesicles from contaminants [51].

Other sEV purification techniques may also cause similar issues when using plasma. For example, there are reports that density gradient centrifugation is likewise not able to completely separate sEVs from APOB^+^ material (i.e. lipoproteins) [24], while immunocapture of sEVs may lead to significant contamination with soluble non-sEV proteins [52]. Thus, a complete separation of sEVs from contaminants of plasma may be an arduous task due to the nature of the plasma samples. A solution can be provided by the use of multiple isolation techniques in combination [34,38] which can result in a purer population of sEVs. However, the yield may be reduced by this approach and the variability is expected to increase due to the requirement of long procedures involving multiple steps. Newer techniques such as field flow fractionation have been reported to achieve good separation of sEVs, and appear promising, but require expensive, specialised equipment, and are relatively low throughput [44].

Our findings are also relevant for experiments where sEVs are isolated from serum-supplemented conditioned medium. As an example, supplementation of medium with 5% FBS (exosome-depleted) will introduce 2.5 ml pure serum into 50 ml of conditioned medium. This may represent a significant problem for SEC-sEV isolations. Typically, an ultrafiltration step is performed before SEC of conditioned medium. Ultrafiltration devices (e.g. Vivaspin, Amicon) concentrate lipoproteins along with the sEVs and we have previously shown that proteins lower than the membrane cut-off of the ultrafiltration units may also be retained [37]. Given the large volumes of conditioned medium normally used for sEV isolation [34], purity will be reduced even further and subsequent analysis will be confounded by the presence of serum-derived material. We argue that the full potential of SEC for sEV isolation is only realised when using serum-free cell culture medium or biological fluids that contain low levels of lipoproteins and protein, such as urine. In fact, a recent comparative study showed that UC-sEVs and SEC-sEVs isolated from serum-free medium conditioned by cardiomyocyte progenitor cells have similar yields and purities, but the SEC-sEVs had increase functionality in terms of activating Erk1/2 in target cells [32]. Similarly, other reports indicate that higher yields and better purity EVs can be isolated by SEC of Opti-MEM™ serum-free medium conditioned by neuroblastoma cells [30]. Therefore, when using serum-free conditions, SEC may provide vesicles with superior purity than UC.

An alternative method of sEV isolation that is quite popular due to its simplicity is precipitation. However, this technique is generally not recommended due to the very low purity achieved [25,33,45,52]. For example, in a recent study, the protein content of the EVs precipitated from 1 ml serum was reported to be 20 mg [18], which is ~20,000 times more than the theoretical protein contained within 10^10^ exosomes [7], and close to the total protein content of serum (~70 mg/ml [53,54]). Therefore, although these serum-derived EVs were found to promote endothelial proliferation, migration and tube-formation [18], their compromised purity may mask any true EV effects.

A series of functional studies related to angiogenesis have been performed using EVs derived from vascular cells or isolated blood cells such as endothelial cells, platelets, leukocytes and erythrocytes (reviewed in [39]). Few reports, however, have specifically addressed the question of whether blood plasma EVs have effects on angiogenesis [17,18]. In the current study, both UC-sEVs and SEC-sEVs promoted migration of endothelial cells and the effects were generally more pronounced with pooled SEC-sEV samples. Angiogenic factor profiling indicated that a variety of potential promigratory molecules are contained within the isolates. UC-sEVs and SEC-sEVs demonstrated very similar profiles with endothelin-1 being the most abundant proangiogenic factor in the samples. Endothelin-1 is known to promote migration and Matrigel invasion of HUVECs, seemingly through actions on ETB receptor [46]. It also increases matrix metalloproteinase expression in endothelial cells and, intriguingly, can have synergistic activities with other proangiogenic factors such as VEGF [46]. The latter can be of importance since VEGF was also present in the sEV samples. Since we also showed that UC-sEVs and SEC-sEVs could be contaminated with soluble protein, we cannot exclude the possibility that endothelin-1 or other potential mediators were co-isolated with the sEVs rather than being present within them. Future extensive studies are required to investigate this possibility, e.g. by western blotting to confirm the presence of endothelin-1 in the isolates and immunolabelling of sEV isolates for TEM imaging and confirmation of endothelin-1 presence within the sEVs.

The main aim of this study was to specifically address the question of whether different normalisation approaches result in discrepant functional outcomes. Intriguingly, HUVEC migration was not proportional to the quantity of the administered CD81 indicating that effects may not be entirely mediated by sEVs. Given the marked contamination we observed in SEC-sEVs relative to UC-sEVs, we argue that contaminating factors (either activating or inhibiting migration) may play a role. Considering our findings and the aforementioned studies, determination of the factors responsible for the promigratory effects and confirmation of their presence within sEVs may be a burdensome task. Importantly, however, in our study, normalisation of UC-sEVs and SEC-sEVs treatments to particle number provided very similar migration effects, while normalisation to total protein content showed significantly higher migration in SEC-sEV group. This discrepancy in the functional outcomes may be further complicated by the recovery rates of sEVs if ultrafiltration units are used to concentrate the samples [55]. Therefore, we suggest that treatment normalisation should be justified, and preferably multiple matching including total protein, particle number and sEV-specific protein content should be performed. Further experimentation may be useful including dose-response relationships accounting for the protein, particle or sEV marker content of UC-sEVs alone and providing reference values for future studies. Detailed dissection of the effects of single SEC fractions will also be helpful in determining whether/which SEC fractions should be pooled for a functional experiment.

Overall, our findings indicate that UC generally isolates blood plasma sEVs of better purity compared to SEC, despite a higher yield of sEVs achieved by SEC. Our functional data on endothelial cell migration suggested that isolation method can have great impact on functional outcomes. Co-isolation of soluble protein and lipoproteins with sEVs when using plasma/serum or other fluids containing blood products may impede interpretation of experimental findings, and the use of a combination of isolation techniques may help overcome these issues.
