# Supplementary material for: Comparison of small extracellular vesicles isolated from plasma by ultracentrifugation or size-exclusion chromatography: yield, purity and functional potential
Source: J Extracell Vesicles. 2018 Dec 28;8(1):1560809. doi: 10.1080/20013078.2018.1560809 (PMC6327926; doi:10.1080/20013078.2018.1560809)
Supplement: Supplemental Material [file ZJEV_A_1560809_SM2687.doc]

Supplemental Material

# Quality of Extracellular Vesicle Images by Transmission Electron Microscopy is Operator and Protocol dependent

L. G. Rikkert^a,b,c*^, R. Nieuwland^b,c^, L. W. M. M. Terstappen^a^ and F. A. W. Coumans^c,d^

*^a^Department of Medical Cell BioPhysics, University of Twente, Enschede, Netherlands; ^b^Amsterdam UMC, University of Amsterdam, Laboratory of Experimental Clinical Chemistry, Amsterdam, Netherlands; ^c^Amsterdam UMC, University of Amsterdam, Vesicle Observation Center, Amsterdam, Netherlands; ^d^Amsterdam UMC, University of Amsterdam, Department of Biomedical Engineering and Physics, Amsterdam, Netherlands*

*Corresponding author: Linda Rikkert
l.g.rikkert@amc.uva.nl

# Supplemental Data

**Supplemental Data 1.** Literature search string.

Search performed in Web of Science on November 22 2016:

**TOPIC:** (exosome) *OR* **TOPIC:** (extracellular vesicle) *OR* **TOPIC:** (cell-derived vesicle) *OR* **TOPIC:** (cell-derived microparticle) *AND* **TOPIC:** (transmission electron microscopy) **Refined by: DOCUMENT TYPES:** (ARTICLE ) **Timespan:** 2011-2016. **Indexes:** SCI-EXPANDED, SSCI, A&HCI, ESCI.

# Supplemental Figures and Tables

## Supplemental Figures

#
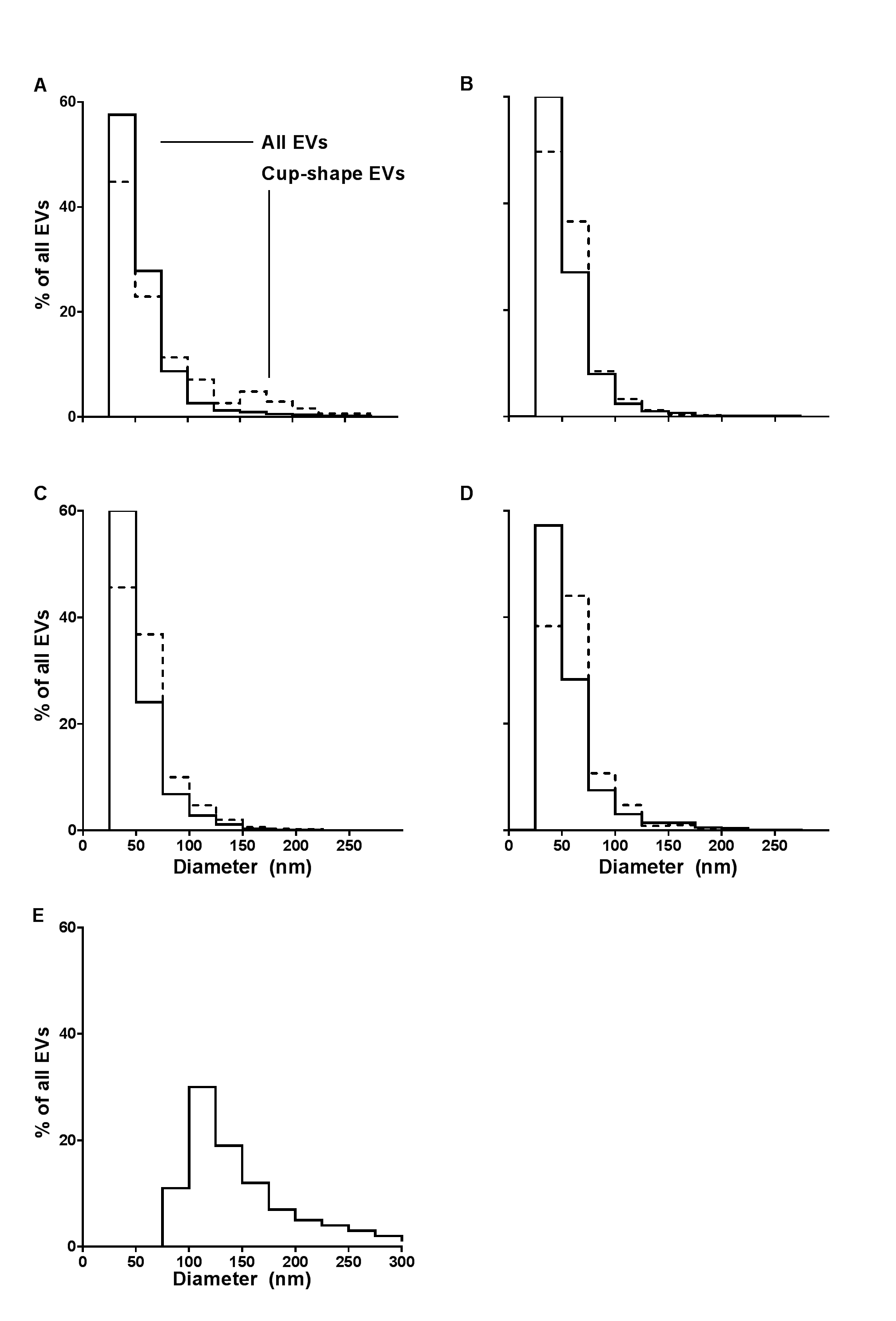


**Supplemental Figure 1. EV diameter histograms by TEM and NTA.** Panels A-D correspond to protocols A-D. Histograms were normalized to 100% area under the curve, and have bin width 25 nm. Solid lines show total EVs, dashed lines cup-shape EVs. The diameter histograms have substantial overlap, and for protocols A-D respectively the median EV diameter was 47, 46, 44, 46 nm. The maximum observed diameter was 392, 292, 219, and 267 nm for protocols A-D respectively. The observed differences in size may be random because the number of EVs >150 nm were 54, 16, 7 and 66, out of a total of 1496-2619 EVs. The differences in diameter may be explained by differences in shrinkage or flattening of EVs, and possibly a difference in the captured population of EVs. No EVs larger than 500 nm were observed, and by definition we did not include any EVs smaller than 30 nm. Panel E shows the particle size distribution of the urinary EV sample (100x diluted in PBS) detected by nanoparticle tracking analysis (NS500, Nanosight, UK). Particle size distribution were derived from 30 measurements of 10 seconds at camera level 14.


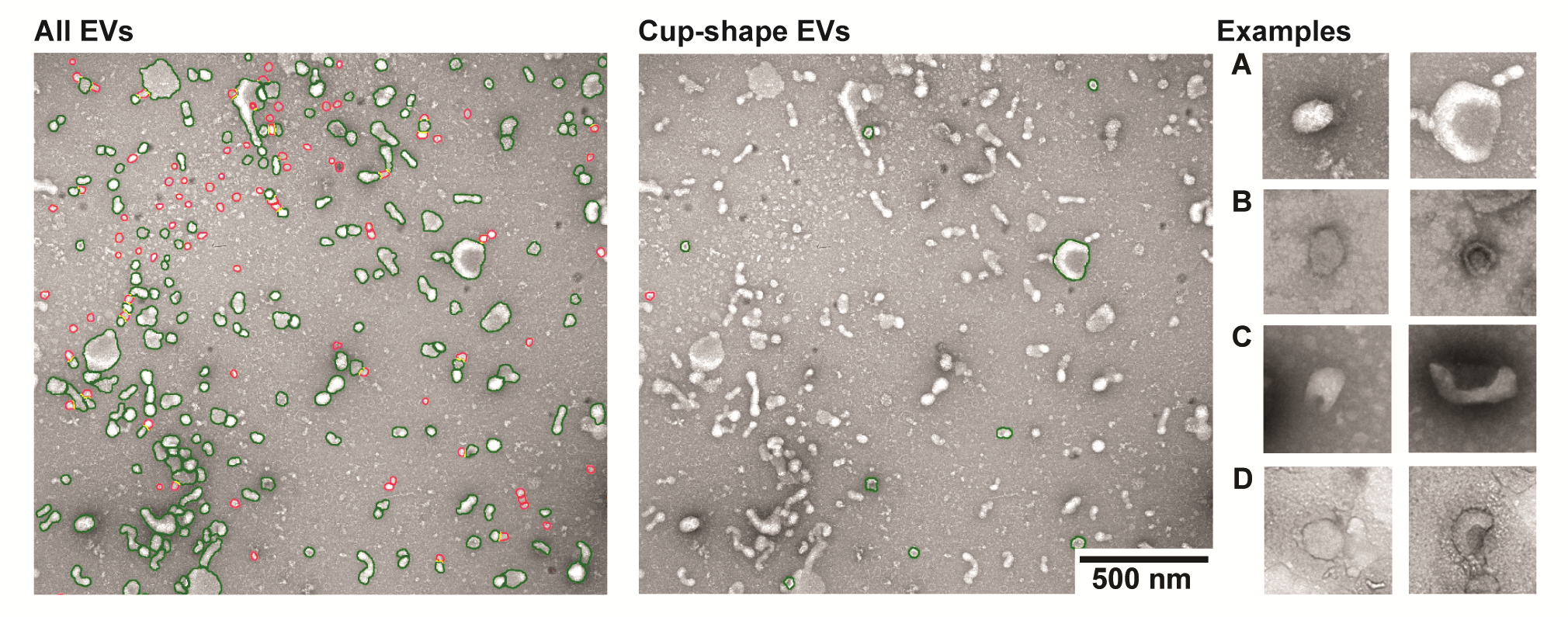


**Supplemental Figure 2. EV selection and automatically determined contours for TEM preparation protocols A-D.** All EVs recognized by the selection tool were manually selected. The images were also analyzed by selection of cup-shape EVs. The green outline shows the contour determined by an automated script, which is used to determine the diameter of each particle. Identified EVs which were smaller than 30 nm were excluded from the analysis (pink outline). Examples of selected cup-shape EVs and excluded non cup-shape particles are shown on the right for each protocol. Scale bar represents 500 nm for the overview images, the examples are 250 x 250 nm.

#
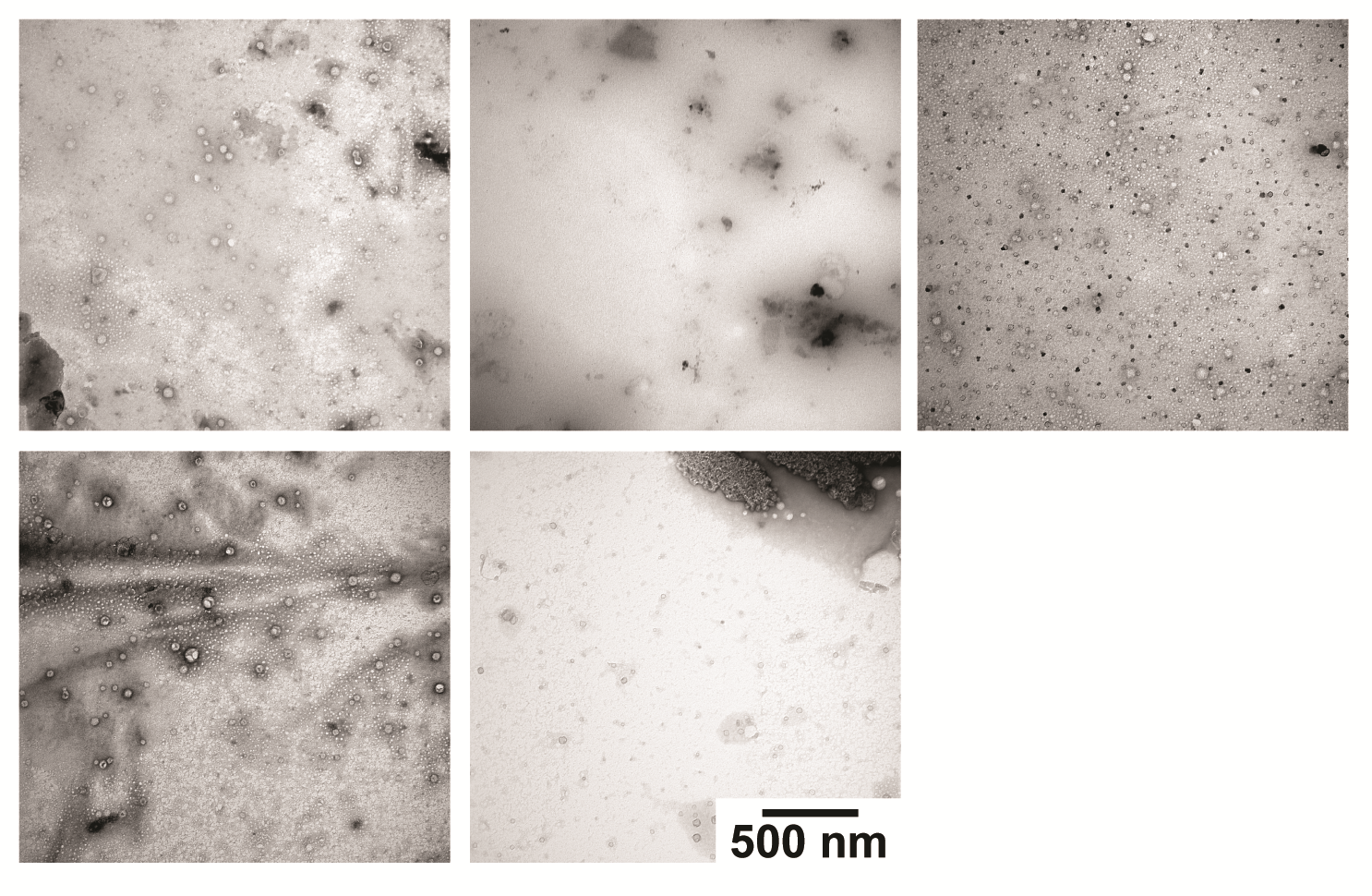


# Supplemental Figure 3. TEM images of plasma after size exclusion chromatography obtained with protocol A at predefined locations. EVs are visible on TEM image of plasma sample after size exclusion chromatography. Scale is the same for all images, bar represents 500 nm.

## Supplemental Tables

**Supplemental Table 1**. Summary data obtained by four TEM preparation protocols.

| Protocol | A | B | C | D |
| --- | --- | --- | --- | --- |
| Total EVs / image | 168 (30) | 100 (37) | 100 (52) | 175 (32) |
| Cup-shape EVs / image | 21 (41) | 32 (80) | 44 (54) | 48 (30) |
| Percentage cup-shape | 14 (58) | 29 (61) | 46 (30) | 28 (22) |
| Image quality score | 3.0 (29) | 2.5 (47) | 2.8 (27) | 2.4 (24) |
| Background quality score | 3.4 (29) | 3.4 (33) | 2.8 (34) | 2.2 (50) |

Mean value shown with CV in parenthesis. Image quality and background quality score can range from 1 (poor) to 5 (excellent).
